# Familial glucocorticoid deficiency presenting with generalized hyperpigmentation in an Egyptian child: a case report

**DOI:** 10.1186/1752-1947-6-110

**Published:** 2012-04-16

**Authors:** Kotb A Metwalley, Hekma S Farghaly

**Affiliations:** 1 Pediatric Endocrinology Unit, Department of Pediatrics, Faculty of Medicine, Assiut University, Assiut, Egypt

## Abstract

**Introduction:**

Familial glucocorticoid deficiency, or hereditary unresponsiveness to adrenocorticotropic hormone, is a rare autosomal recessive disease characterized by glucocorticoid deficiency in the absence of mineralocorticoid deficiency. It may present in infancy or early childhood with hyperpigmentation, failure to thrive, recurrent infections, hypoglycemic attacks and convulsions that may result in coma or death. Here, we report the case of an 18-month-old Egyptian boy with familial glucocorticoid deficiency.

**Case presentation:**

An 18*-*month*-*old Egyptian boy was referred to our institution for evaluation of generalized hyperpigmentation of the body associated with recurrent convulsions; one of his siblings, who had died at the age of nine months, also had generalized hyperpigmentation of the body. The initial clinical examination revealed generalized symmetrical deep hyperpigmentation of the body as well as hypotonia, normal blood pressure and normal male genitalia. He had low blood glucose and cortisol levels, normal aldosterone and high adrenocorticotropic hormone levels. Based on the above mentioned data, a provisional diagnosis of familial glucocorticoid deficiency was made, which was confirmed by a molecular genetics study. Oral hydrocortisone treatment at a dose of 10 mg/m^2^/day was started. The child was followed up after two months of treatment; the hyperpigmentation has lessened in comparison with his initial presentation and his blood sugar and cortisol levels were normalized.

**Conclusion:**

Familial glucocorticoid deficiency is a rare, treatable disease that can be easily missed due to nonspecific presentations. The consequences of delayed diagnosis and treatment are associated with high rates of morbidity and mortality.

## Introduction

Familial glucocorticoid deficiency (FGD) is a rare autosomal recessive disease resulting from resistance to the action of adrenocorticotropic hormone (ACTH) on the adrenal cortex, which leads to isolated glucocorticoid deficiency with normal mineralocorticoid secretion [1]. FGD is a rare disease; its exact incidence is unknown and only isolated case reports are documented. FGD (OMIM 202200) was first described by Shepard *et al. *in 1959 [2]. Pathologic evaluation of children affected with this disorder reveals that the zona glomerulosa of the adrenal glands is well preserved while the zona fasciculata and zona reticularis are markedly atrophic. These changes are accompanied by low plasma cortisol concentrations because the zona fasciculata is primarily responsible for glucocorticoid production. Low circulating serum cortisol results in a lack of feedback inhibition to the hypothalamus; markedly increased ACTH levels are often observed. Because the zona glomerulosa is generally well preserved, mineralocorticoid production is usually unaffected. Plasma renin and aldosterone concentrations are usually within the reference range in the baseline state and demonstrate normal variability on salt restriction [3].

FGD may be caused by mutations in the gene of the ACTH receptor (melanocortin 2 receptor; MC2R), classified as FGD type 1, or by mutations in the melanocortin-2 receptor accessory protein (MRAP), classified as FGD type 2. Mutations of the ACTH receptor (type 1) account for 25% of FGD cases, while FGD type 2 accounts for approximately 15% to 20% of FGD cases [4]. These mutations may affect ACTH signal transduction, expression of the ACTH receptor or differentiation of the adrenal cortex [5].

## Case presentation

An 18-month-old Egyptian boy was referred to our pediatric endocrinology clinic for evaluation of generalized hyperpigmentation of the skin associated with weakness, vomiting and recurrent convulsions. He was born to consanguineous parents after an uneventful pregnancy with a birth weight of 3450 g. He was the fourth child of the family; one of his siblings, who had died of an unknown cause at the age of nine months, had generalized hyperpigmentation of the body, recurrent convulsions and mental retardation. His other siblings were reported to be healthy.

**Figure 1:**
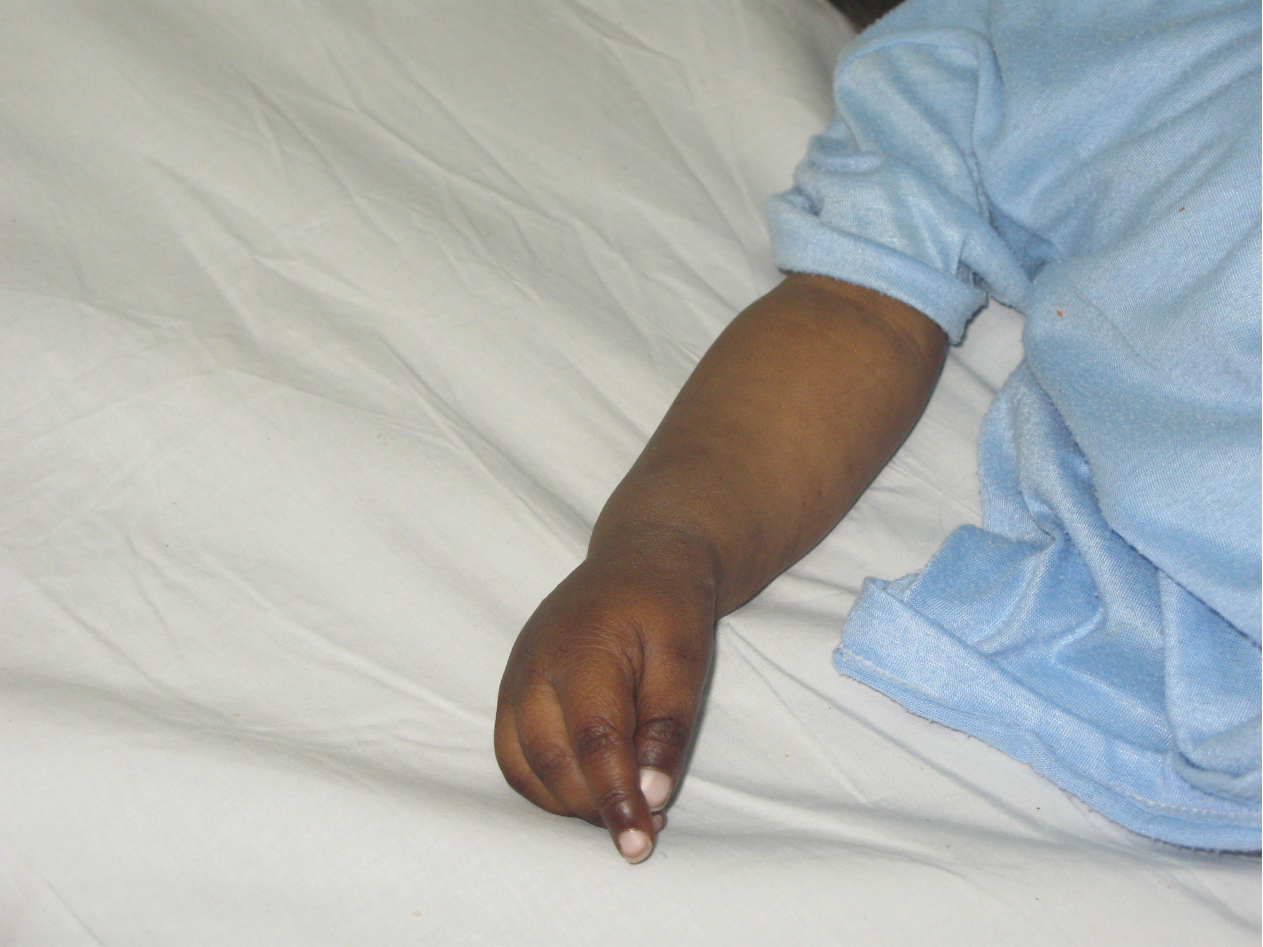
**Hyperpigmentation of the upper limb before treatment**.

**Figure 2:**
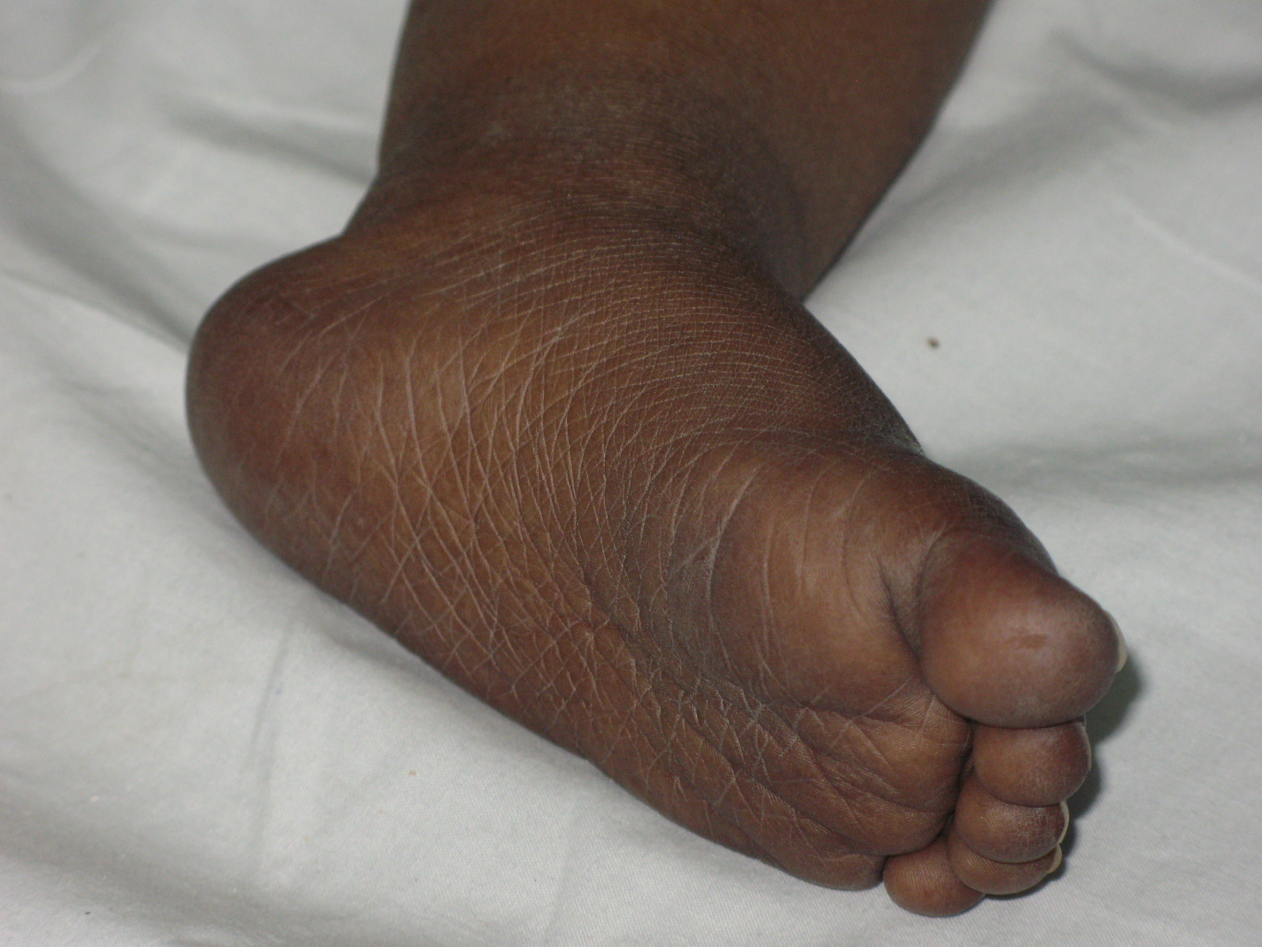
**Hyperpigmentation of the foot before treatment**.

After the first few weeks of the baby's life, his mother noticed a generalized black coloration of his skin. His parents also reported that he was darker than his sister and brother. At the ages of 10 and 11 months he had two attacks of a febrile brief generalized tonic-clonic convulsion. The etiology of the convulsions had not been evaluated as the parents did not seek medical advice at that time. On examination, our patient was able to speak only a few words and walk with support. His weight was in the 5^th ^percentile while his length and head circumference were in the 50^th ^percentile. His blood pressure was 90/60 mmHg. Unlike his fair-skinned parents, he had generalized symmetric deep hyperpigmentation of the body, especially of his elbows, knees, hands, feet and buccal mucosa (Figures 1 and 2), as well as hypotonia. He had no dysmorphic features. Examination of his external genitalia revealed a penis of normal length and bilaterally palpable testes in his scrotum. There were no signs of alacrima or achalasia. Other examination findings were unremarkable.

His blood chemistry results were: glucose, 48 mg/dL; sodium, 140 mmol/L; potassium, 4 mmol/L; chloride, 109 mmol/L; alanine transaminase, 28 IU/mL; and aspartate transaminase, 30 IU/mL. His total blood count was normal. His cortisol level was 0.6 μg/dL (normal range: 2.8 to 23 μg/dL) and ACTH, 708 pg/mL (normal range: 6 to 48 pg/mL). An ACTH stimulation test did not cause a rise in his cortisol level. His 17-OH progesterone level was 0.01 ng/mL (normal range: 0.03 to 0.9 ng/mL); androstenedione, 0.01 ng/mL (normal range: 0.1 to 0.17 ng/mL), dehydroepiandrosterone, 3 ng/mL (normal range: 50 to 480 ng/mL); plasma rennin activity, 57 ng/mL/hour (normal range: 2.35 to 37 ng/mL/hour); aldosterone, 801 pg/mL (normal range: 50 to 900 pg/mL).

His bone age, evaluated by an X-ray of his left hand and wrist according to the Greulich-Pyle atlas, was equivalent to two years. Adrenal ultrasonography revealed that both his adrenal glands were small; his left was 6 × 8 mm and right 7 × 8 mm in diameter. Computerized tomography of his brain was normal. A molecular genetics study revealed normal ACTH receptor gene sequences, excluding FGD type 1, and a homozygous deletion (c. 111+1delG) in intron 3 of the MRAP gene was detected. Based on these findings, a diagnosis of FGD type 2 was made. Oral hydrocortisone treatment at a dose of 10 mg/m^2^/day was started.

**Figure 3:**
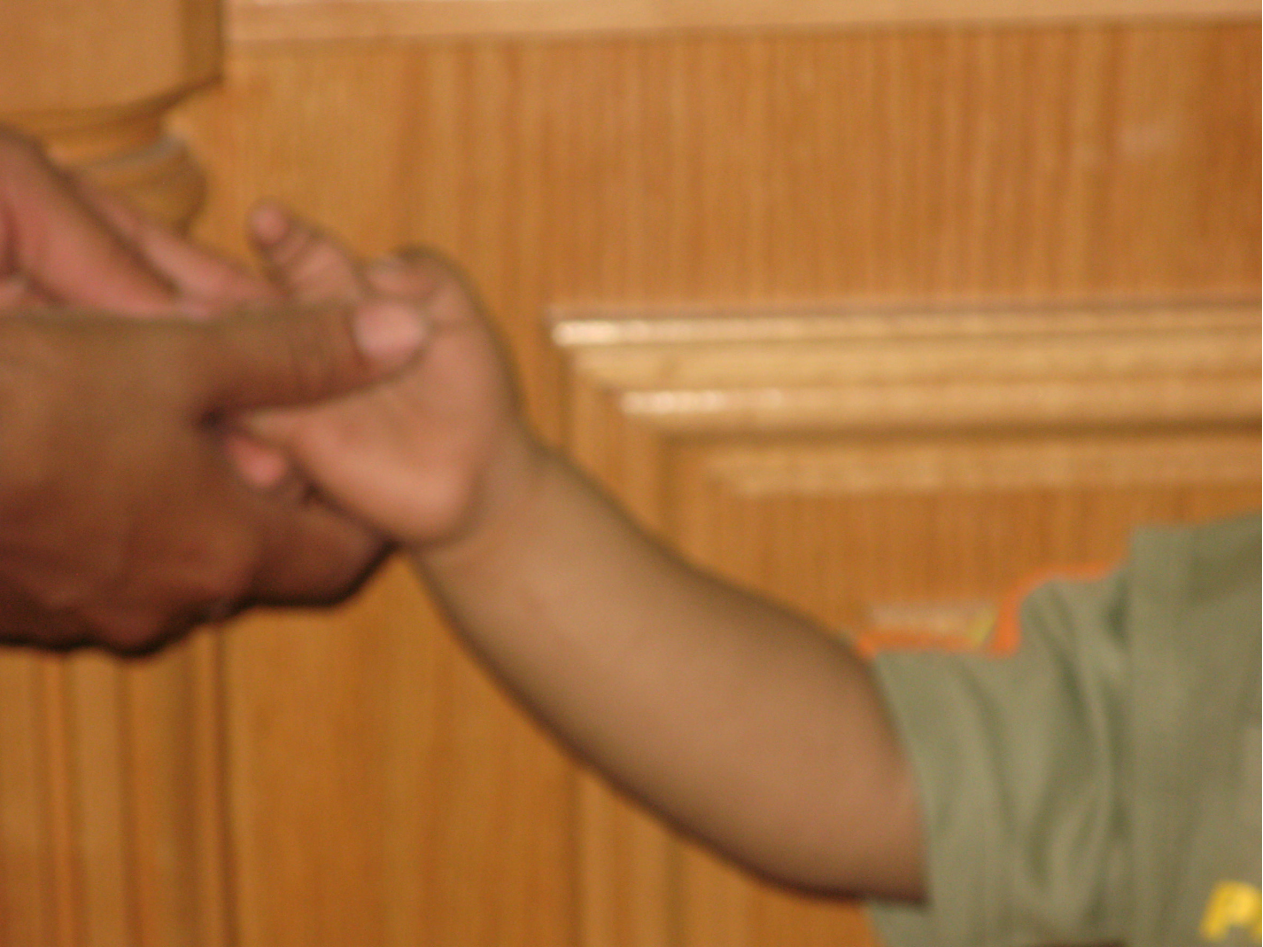
**Improvement in hyperpigmentation of the upper limb after treatment**.

**Figure 4:**
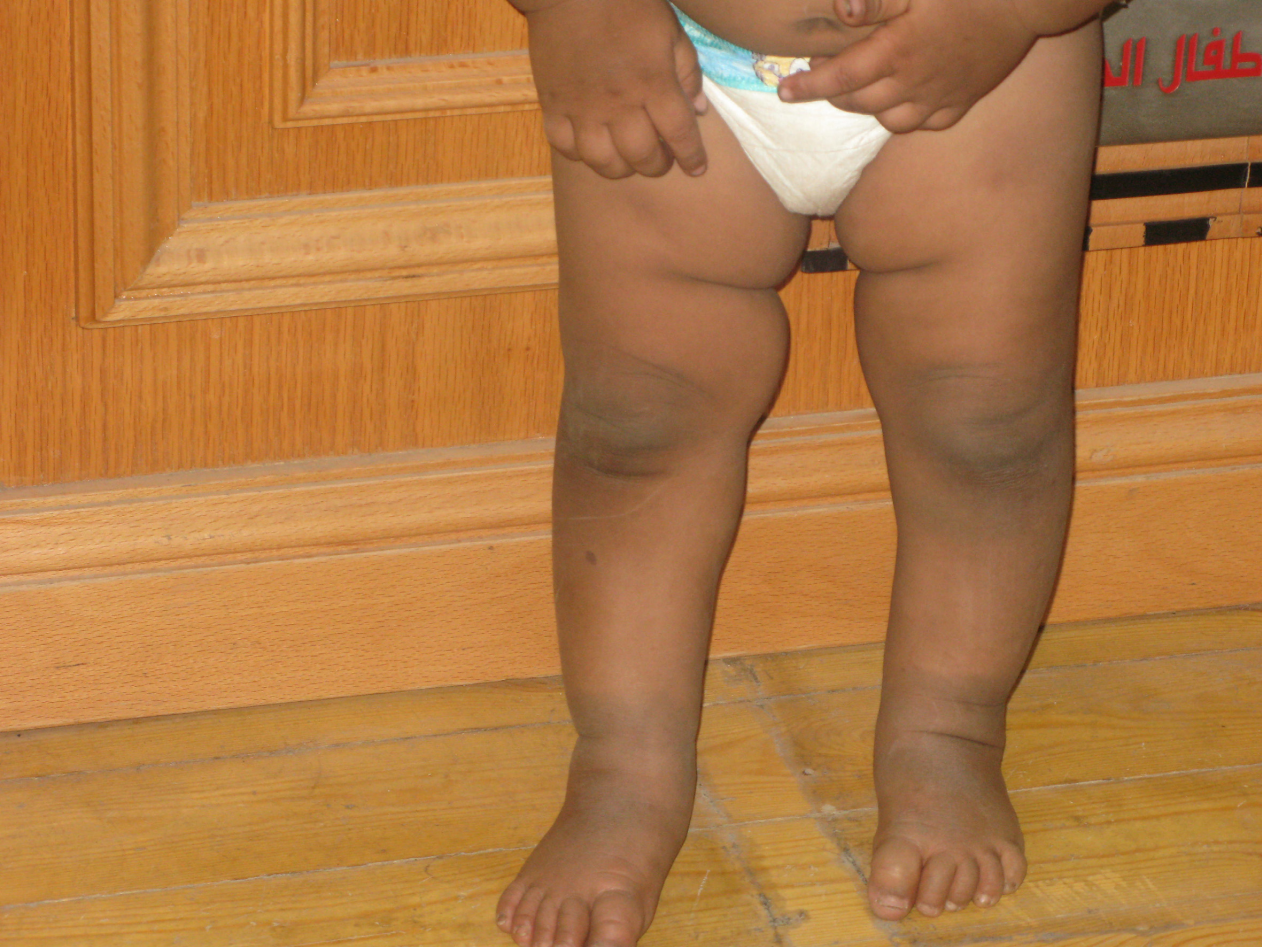
**Improvement in hyperpigmentation of the lower limbs after treatment**.

Our patient was seen two months after starting treatment. His general condition was greatly improved and he was able to walk alone. The hyperpigmentation was less in comparison with his initial presentation (Figures 3 and 4), he had gained 1500 g, and his blood sugar and cortisol levels were normalized while the level of ACTH had dropped to 221 pg/mL.

## Discussion

Unawareness of FGD can lead to delay in diagnosis, not only in the affected patient but also among other members of the family. The disorder can be fatal if proper treatment is not provided. FGD should be distinguished from other causes of adrenal insufficiency, which include congenital adrenal hyperplasia, adrenal hypoplasia, adrenoleukodystrophy, Allgrove syndrome, adrenal hemorrhage, trauma and infections, by taking a thorough history, clinical examination and appropriate investigations [6].

A review of the literature revealed that the age of onset of symptoms in patients with FGD ranges from birth to nine years old. All the symptoms and signs seen in FGD are the result of either hypocortisolemia or elevated ACTH levels, of which our patient had both. Hypocortisolemia may cause weakness, fatigue, weight loss, anorexia progressing to nausea, vomiting, diarrhea, constipation, flank or abdominal pain, hypoglycemia and hypothermia. High ACTH causes increased melanin production, resulting in generalized hyperpigmentation and advanced bone age [7,8]. Bone age was advanced by four months in our patient, which may be attributed to increased ACTH or related pathophysiologic changes [4]. The long-term neurological consequence of FGD can vary from learning difficulties to spastic quadriplegia, which may reflect the severity and number of hypoglycemic episodes during childhood [9]. The most frequent cause of FGD death is undiagnosed glucocorticoid insufficiency. Although this disease is easily treatable when recognized, if left untreated it may be fatal or lead to severe mental disability as a result of recurrent hypoglycemia secondary to glucocorticoid insufficiency.

Our patient was diagnosed with FGD at the age of 18 months; he had two attacks of convulsive episodes prior to the presentation that could be attributed to unrecognized hypoglycemia. Modan-Moses *et al. *[9] reported an Ethiopian infant with FGD, who presented with psychomotor retardation, spastic quadriparesis and microcephaly due to severe hypoglycemic attacks.

Our patient was diagnosed with FGD type 2 by a molecular genetics study, through detection of mutations in the MRAP gene (OMIM 607398). *MRAP*, located at 21q22.1, is an essential cofactor for MC2R expression in certain cell types and seems to have a role in the processing, trafficking or function of MC2R [5]. MRAP has two isoforms, namely MRAPα and MRAPβ, that differentially regulate the function of MC2R [10]. Akin *et al. *[6] reported a Turkish patient with FGD type 2, with a known MRAP mutation. Their patient was the offspring of consanguineous parents and his four siblings had died in the neonatal period, probably due to glucocorticoid insufficiency.

The treatment of FGD is by replacement with hydrocortisone. An oral dose of 10 to 12 mg/m^2^/day in three divided doses is usually sufficient. The suppression of plasma ACTH levels in FGD can be very difficult and should not be used as the goal of treatment. Educating parents and patients on the need to increase hydrocortisone dosages during illness and emergency management with intramuscular hydrocortisone is vital [11]. In addition, replacement with hydrocortisone in children with FGD should not be a contraindication to vaccinate with live attenuated vaccines [12].

## Conclusion

FGD is a rare, treatable disease that can be easily missed due to its nonspecific presentation. The consequences of delayed diagnosis and treatment are associated with high rates of morbidity and mortality. Clinical awareness of this condition is of considerable prognostic and therapeutic significance.

## Abbreviations

ACTH: adrenocorticotropic hormone; FGD: familial glucocorticoid deficiency; MC2R: melanocortin-2 receptor; MRAP: melanocortin-2 receptor accessory protein.

## Consent

Written informed consent was obtained from the patient's parents for publication of this case report and any accompanying images. A copy of the written consent is available for review by the Editor-in-Chief of this journal.

## Competing interests

The authors declare that they have no competing interests.

## Authors' contributions

KA and HS diagnosed, investigated, followed up and managed the patient drafted the manuscript. Both authors read and approved the final manuscript.
